# Discovery and Community Dynamics of Novel ssRNA Mycoviruses in the Conifer Pathogen *Heterobasidion parviporum*

**DOI:** 10.3389/fmicb.2021.770787

**Published:** 2021-11-24

**Authors:** Suvi Sutela, Tuula Piri, Eeva J. Vainio

**Affiliations:** Natural Resources Institute Finland (Luke), Helsinki, Finland

**Keywords:** mycovirus, forest pathogen, boreal forest, virus population, *Lenarviricota*, ambisense virus

## Abstract

*Heterobasidion* species are highly destructive basidiomycetous conifer pathogens of the Boreal forest region. Earlier studies have revealed dsRNA virus infections of families *Curvulaviridae* and *Partitiviridae* in *Heterobasidion* strains, and small RNA deep sequencing has also identified infections of *Mitoviridae* members in these fungi. In this study, the virome of *Heterobasidion parviporum* was examined for the first time by RNA-Seq using total RNA depleted of rRNA. This method successfully revealed new viruses representing two established (+)ssRNA virus families not found earlier in *Heterobasidion*: *Narnaviridae* and *Botourmiaviridae*. In addition, we identified the presence of a recently described virus group tentatively named “ambiviruses” in *H. parviporum*. The *H. parviporum* isolates included in the study originated from experimental forest sites located within 0.7 km range from each other, and a population analysis including 43 isolates was conducted at one of the experimental plots to establish the prevalence of the newly identified viruses in clonally spreading *H. parviporum* individuals. Our results indicate that viral infections are considerably more diverse and common among *Heterobasidion* isolates than known earlier and include ssRNA viruses with high prevalence and interspecies variation.

## Introduction

The existence of viral infections in fungi was first discovered by Hollings (1962) who described virus-associated disease symptoms in the cultivated mushroom *Agaricus bisporus*. Fungal viruses have since then been described in diverse fungal taxa, including Ascomycota and Basidiomycota as well as early diverging fungal lineages such as Chytridiomycota, Blastocladiomycota, Neocallimastigomycota, Zoopagomycota, and Mucoromycota (Ghabrial and Suzuki, 2009; Pearson et al., 2009; Ghabrial et al., 2015; Sutela et al., 2019, 2020; Myers et al., 2020). Mycoviruses that mediate reduced virulence (hypovirulence) in plant pathogenic fungi have been widely studied for potential use as biocontrol agents, which has revealed debilitation associated viruses also in important pathogens of trees, such as *Ophiostoma novo-ulmi*, *Rosellinia necatrix*, and *Heterobasidion* spp. (Kanematsu et al., 2004; Chiba et al., 2009; Sasaki et al., 2016; Vainio et al., 2018a; Kashif et al., 2019). Currently, hypoviruses are used for biocontrol against the Chestnut blight disease caused by *Cryphonectria parasitica* in Europe (Milgroom and Cortesi, 2004; Rigling and Prospero, 2018).

High-throughput sequencing (HTS) studies have recently revolutionized our understanding on the virosphere and revealed numerous novel virus clades that await classification by the International Committee on Taxonomy of Viruses (ICTV). Among these are many new fungal narna-like viruses (Chiapello et al., 2020, 2021; Sutela et al., 2020; Chiba et al., 2021; Jia et al., 2021) and the unclassified virus group tentatively named “ambiviruses” recently detected in asco- and basidiomycetous fungi (Sutela et al., 2020; Forgia et al., 2021; Linnakoski et al., 2021). The accumulating sequence information has also led to the recognition of new host taxa for viruses, for example, plants as hosts for mitoviruses (Nibert et al., 2018) and fungi as hosts of ourmia-like viruses (now classified in the viral family *Botourmiaviridae* in order *Ourlivirales*), and the re-classification of many fungal viruses, for example, the division of family *Narnaviridae* into *Mitoviridae* and *Narnaviridae*.

The *Heterobasidion* species complex includes several highly destructive conifer pathogens of the Boreal forest region (Garbelotto and Gonthier, 2013). These root rot fungi produce basidiospores that infect freshly cut conifer stumps or wounded trees, and the established mycelium may survive for decades, spreading from tree to tree *via* root contacts. *Heterobasidion parviporum* prefers Norway spruce (*Picea abies*) as its host tree and is widely distributed in the Northern hemisphere following the distribution of its primary host (Garbelotto and Gonthier, 2013).

Earlier studies have revealed double-stranded RNA (dsRNA) virus infections in ca. 15–17% of *Heterobasidion* strains (Ihrmark, 2001; Vainio and Hantula, 2016). The most common dsRNA virus in *Heterobasidion* spp. (including *H. parviporum*) is Heterobasidion RNA virus 6, a bisegmented virus in family *Curvulaviridae* (Vainio et al., 2012, 2015a). Members of family *Partitiviridae* are also relatively common and highly diverse among species of *Heterobasidion* (Vainio and Hantula, 2016). The alphapartitivirus HetPV13-an1 from *Heterobasidion annosum* has been associated with reduced host growth and changes in host gene expression (Vainio et al., 2018a), and another alphapartitivirus from *H. parviporum*, HetPV15-pa1, also causes host debilitation (Kashif et al., 2019).

Thus far, only a few *Heterobasidion* strains have been investigated by HTS, and both small RNA deep sequencing and transcriptomics analysis have successfully revealed the presence of mitoviruses not detectable as dsRNA in *H. parviporum* and *H. annosum* (Vainio et al., 2015b; Vainio, 2019). However, RNA-Seq analysis using total RNA depleted of rRNA has not been tested for *Heterobasidion* virus detection prior to this study. Here, we tested whether new viral diversity (particularly novel single-stranded RNA (ssRNA) virus taxa that may be more difficult to detect as dsRNA) could be discovered in *H. parviporum* using this methodology. The investigated isolates originated from experimental forest sites located within 0.7 km range from each other, one of which has been extensively screened for the presence and distribution of dsRNA viruses earlier (Vainio et al., 2015a). The earlier investigation showed the temporal persistence and accumulation of dsRNA viruses of families *Partitiviridae* and *Curvulaviridae* in long-living *H. parviporum* clones, which were revisited in this study to examine the following research questions: (i) are adjacent *Heterobasidion* clones infected with identical ssRNA virus variants suggesting lateral virus transmission; (ii) are ssRNA virus infections temporally stable within *Heterobasidion* clones; (iii) do ssRNA viruses accumulate in *Heterobasidion* clones through time, leading to increase in viral co-infections in single isolates.

## Materials and Methods

### The Origin and Isolation of *H. parviporum* Strains

Most of the *H. parviporum* strains included in the RNA-Seq analyses were isolated from the Ruotsinkylä research forest in Tuusula, situated approximately 30 km north of Helsinki. They originated from five different experimental plots (3R, 4R, 7R, RKU, RKU3), all of which were located within ca. 700 m distance from each other. More detailed site characteristics for RKU are described in Piri and Korhonen (2007) and those for 3R, 4R, and 7R in Piri and Korhonen (2008), where 3R = plot T1, 4R = plot T3, and 7R = plot U4. All *Heterobasidion* isolates subjected to RNA-Seq (except RKU172) contained dsRNA (data not shown) as revealed by cellulose chromatography analysis (Morris and Dodds, 1979; Tuomivirta and Hantula, 2003a).

LAP3.3.2^∗^5 is a *H. parviporum* strain infected with five viruses (HetPV4-pa1, HetRV6-pa32, HetPV13-an1, HetPV16-an1, and HetPV2-pa1) by laboratory pairing experiments as described by Hantula et al. (2020). Two of the viruses hosted by this isolate originated from the Ruotsinkylä research forest: HetPV4-pa1 originally hosted by isolate RT3.49C from the 4R plot (Vainio et al., 2011) and HetPV2-pa1 from the 7R plot (Vainio et al., 2015a).

**FIGURE 1:**
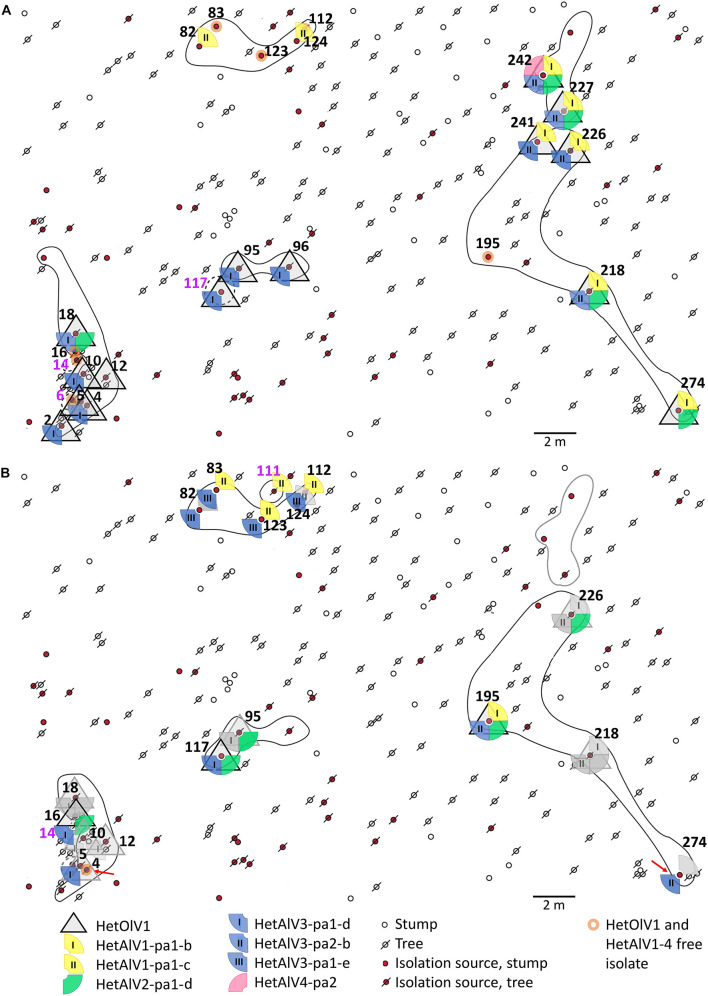
Spatial distribution of Heterobasidion ourmia-like virus 1 (HetOlV1), Heterobasidion ambi-like viruses 1–4 (HetAlV1–4), and *H. parviporum* isolates in 2005 **(A)** and 2012 **(B)**. The isolation sources refer to individual stumps and trees with *H. parviporum* infection 2005 and resampled 2012. The sampling points of *H. parviporum* isolation sources included in the analyses are indicated with numbers (black font = clone includes more than single isolate; purple = clone includes only one isolate) and clones marked with circles (solid line = heterokaryon; dashed line = homokaryon; black line = viable clone; gray line = clone not found in 2012). Red arrows indicate viral losses. Symbols in gray (**B**, 2012 situation) indicate that the virus content remained the same as in 2005, and colorful symbols indicate virus gains in the isolation source.

The *H. parviporum* population utilized in the RT-PCR screening of mycoviruses newly discovered in the present study originated from a naturally regenerated Norway spruce stand 7R of 875 m^2^ located in Ruotsinkylä (60°22′05″ N, 24°59′53″ E), southern Finland (see Vainio et al. (2015a) for detailed site characteristics). The isolation of *H. parviporum* strains took place in 2005 (Piri and Korhonen, 2008) and 2012 (Vainio et al., 2015a), and the clonal structure of the *H. parviporum* isolates had been determined previously using vegetative incompatibility reactions and microsatellite markers (Vainio et al., 2015a). The isolates analyzed in the present study (*N* = 43) are presented in Figure 1 and Table 3.

### Maintenance and Storage of *H. parviporum* Cultures

The *H. parviporum* strains utilized in the study were stored for long-term in agar slides (2% malt extract medium, MEA) in cryotubes and kept in a cold room (4°C). The long-term preservations were renewed (by cultivating the strain on fresh MEA plates for ∼2 weeks) every time the particular strain was accessed. The estimated cultivation count is three for the oldest strains (isolated in 2003) and three or less for the *H. parviporum* strains isolated after 2003.

### Total RNA Isolation and High-Throughput Sequencing

The presence of mycoviruses in *H. parviporum* isolates was examined using RNA sequencing (RNA-Seq). The cDNA library named 7Het contained eight *H. parviporum* isolates (LAP3.3.2^∗^5, RT3.45, 3R4, RKU3.3.34, 4R1, RKU172, RKU3.2.124, and RKU3.1.25), one *H. annosum* isolate, and one *Oidiodendron maius* isolate. Here, we report the results for the *H. parviporum* strains.

#### Cultivation of the Fungal Isolates and Extraction of Total RNA

The *H. parviporum* isolates were cultivated for 6–9 days in 1.5% malt extract on an orbital shaker (120 rpm) at 23°C. The collected mycelia was freeze-dried for 3 days and kept in −80°C until the total RNA was isolated using Spectrum Plant Total RNA Kit (Sigma-Aldrich) as described by Sutela et al. (2020) with 800 μl of Lysis Solution and 25–33 mg of pulverized fungal mycelia. The quality and quantity of extracted total RNA were examined using NanoDrop (Thermo Scientific) and by running an aliquot of each RNA on an agarose gel.

#### Library Construction and Bioinformatics Pipeline

A pooled RNA sample, composed of 0.5 μg total RNA of each fungal isolate, was sent to Macrogen Korea for library preparation and HTS. The library was generated using the TruSeq Stranded Total RNA Library prep kit with Human/Mouse/Rat Gold for rRNA removal (Illumina), and Novaseq (Illumina) was utilized in the production of 101 bp pair-end reads (100 M reads/sample).

The raw reads were pre-processed with Trimmomatic (Bolger et al., 2014) and subsequently Trinity 2.8.4 (Grabherr et al., 2011) conducted the *de novo* assembly in R (R Development Core Team, 2011). Host specific contigs were determined with BlastX (*e*-value 10e-5) using a database (db) generated by combining translated CDS of hosts (Olson et al., 2012; Kohler et al., 2015; Martino et al., 2018). The contigs showing significant similarity were omitted, and subsequently, BlastX runs were performed against the custom-made viral protein dbs. Contigs showing similarity with viral proteins were then examined using BlastN and BlastX searches against complete nucleotide and non-redundant protein sequences at NCBI. Putative virus contigs were further studied in Geneious 10.2.6 (Biomatters Ltd.). The raw reads were mapped against putative viral contigs in order to examine the accuracy of the Trinity contigs, the coverage, and sequence polymorphism and to produce full-length virus segments.

#### Identification of Host Isolates and Validation of *in silico* Assembled Virus Genomes

The *H. parviporum* isolates included to the HTS were grown in the dark at RT on MOS agar plates covered with cellophane membranes. The nucleic acids were isolated from mycelia using phenol-chloroform extraction and PEG 6000 precipitation as previously described (Vainio et al., 2013). RevertAid Reverse Transcriptase (Thermo Fisher Scientific) was used in the conversion of RNA to cDNA with a 5 min initial denaturation step at 99°C. The hosts of putative viruses were determined with standard RT-PCR using primer pairs specific for viral contigs (Supplementary Table 1) and DreamTaq DNA Polymerase (Thermo Fisher Scientific). The possibility of genomic integration of putative mycoviruses was examined with standard PCR using identical reaction conditions as with the RT-PCR. The DNA of *H. parviporum* strains were isolated as described in Vainio et al. (1998).

### Characterization of Virus Genomes

In order to determine the sequence differences between virus variants hosted by different *H. parviporum* isolates present in the 7Het library, Sanger sequencing was conducted. This also allowed us to determine whether all strains and variants representing a virus species could be detected in a mixed virus pool based on read mapping and single nucleotide polymorphism (SNP) detection. For SNP determination, the mapping of reads to virus strains and variants or contigs was conducted with Geneious RNA assembler with the loose mapping criteria (medium-low sensitivity allowing 20% as maximum mismatches per read). Variant call parameters for virus sequences are presented in Supplementary Table 2. Moreover, the 5′ and 3′ untranslated regions (UTRs) of selected novel viruses were determined as the ends of mycovirus segments may not be covered by the *in silico* contig assembly. All the Sanger sequencing was conducted at Macrogen Europe, and Geneious 10.2.6 (Biomatters Ltd., New Zealand) was utilized in the examination of sequences and assembly of mycovirus sequences.

#### Sequence Determination of the Novel Mycoviruses

If a novel virus was hosted by more than one *H. parviporum* isolate, its complete genome or complete CDS was determined by Sanger sequencing at least from one isolate. The RT-PCRs were conducted with Phusion High-Fidelity DNA Polymerase (Thermo Fisher Scientific) and virus contig specific primer pairs (Supplementary Table 1). All sequence positions were determined twice from two PCR reactions.

The cellulose chromatography method (Morris and Dodds, 1979; Tuomivirta and Hantula, 2003a) was utilized to extract dsRNA used for the single primer amplification technique of Lambden et al. (1992). The dsRNA were either run on an agarose gel and ligated with T4 adapter (Tuomivirta and Hantula, 2003b), or total dsRNA was first ligated with T4 adapter and subsequently run on an agarose gel. The RNAid kit (Bio101, Carlsbad) was used to purify the dsRNA after the agarose gel electrophoresis. The initial denaturation step of reverse transcription was 3 and/or 6 min at 99°C. Maxima H Minus Reverse Transcriptase (Thermo Fisher Scientific) and/or RevertAid H minus Reverse Transcriptase (Thermo Fisher Scientific) were utilized in the reverse transcription at 55 or 50°C, respectively. The 5′ and 3′ UTR regions were amplified with Phusion High-Fidelity DNA Polymerase (Thermo Fisher Scientific) in a reaction with a primer specific for T4 RNA adapter and a primer specific to the 5′ or 3′ end of the mycovirus. See Supplementary Table 1 for the primer information. PCR products were sequenced at Macrogen Europe, and each sequence position was covered by at least two Sanger sequences. Geneious 10.2.6 (Biomatters Ltd., New Zealand) was utilized in the generation of the assembly.

#### Determination of the Different Virus Variants by Sanger Sequencing

The nucleic acid isolation and reverse transcription was performed as described previously. The RT-PCRs were conducted with Phusion High-Fidelity DNA Polymerase (Thermo Fisher Scientific) and virus contig specific primer pairs producing partial CDS regions (Supplementary Table 1). All mycovirus sequences were Sanger sequenced in both directions.

### Phylogenetic Analysis

Phylogenetic trees were constructed using predicted RNA-dependent RNA polymerases (RdRPs) of mycoviruses. The RdRPs of related viruses were retrieved from the GenBank and aligned using MAFFT v7.450 with Blosum45 substitution matrix. The evolutionary history was inferred by using the Maximum Likelihood method in MEGA X (Kumar et al., 2018). Detailed information on the substitution model and parameters utilized are described in figure legends.

### Prevalence of Virus Infections in the *H. parviporum* Clones at the 7R Forest Plot

*Heterobasidion parviporum* strains isolated in 2005 and 2012 (Figure 1; Table 3; Vainio et al., 2015a) representing nine different *H. parviporum* clones were examined for the presence of novel viruses identified in the present study by HTSs. The virus specific primer pairs (Supplementary Table 1) were used in a standard RT-PCR with DreamTaq DNA Polymerase (Thermo Fisher Scientific). To obtain a longer PCR product for sequencing, the virus infected isolates were selected for a second RT-PCR conducted using Phusion High-Fidelity DNA Polymerase (Thermo Fisher Scientific) and/or DreamTaq DNA Polymerase (Thermo Fisher Scientific) and a virus specific primer pair. The RT-PCR products were Sanger sequenced at Macrogen Europe and examined using Geneious 10.2.6 (Biomatters Ltd., New Zealand).

## Results

### Detection of Mycoviruses in the RNA-Seq Library

The *H. parviporum* RNA-Seq library 7Het was composed of ∼118 million paired-end reads (SRA ID: SRR14842285). Using a bioinformatics pipeline based on Trinity *de novo* assembly (of 116,046 contigs), we were able to identify a new narna-like virus and a new ourmia-like virus, which were the first putative members of families *Botourmiaviridae* and *Narnaviridae* described in species of *Heterobasidion* (Table 1). We also detected four novel viruses belonging to a recently discovered virus group named “ambiviruses” (Sutela et al., 2020; Forgia et al., 2021). Moreover, we discovered new strains and variants of previously described species of mito- and orthocurvulaviruses (Vainio et al., 2012; Vainio, 2019) and a new putative species of virus family *Partitiviridae* (Tables 1, 2).

**TABLE 1:** Novel mycoviruses detected in our *H. parviporum* isolate collection.

**Mycovirus**	**Segment**	**GenBank ID**	**Host strain**	**Length (nt)**	**GC %**	**Accession with the highest identity^a^**	***e*-value**	**Identity %**	**Query cover**	**Mapping reads^b^**	**Average depth^c^**
Heterobasidion narna-like virus 1 (HetNlV1)	RNA1	MZ502381	RKU172	3,983	54.0	Plasmopara viticola lesion associated orfanplasmovirus 1^d^	3e-73	29.9%	72%	91,168	2,312
	RNA2	MZ502382	RKU172	3,960	56.0	Insect narna-like virus 1^e^	7e-5	30.8%	9%	29,203	734
Heterobasidion ourmia-like virus 1 (HetOlV1)	RNA1	MZ502383	RKU3.2.124	2,532	49.7	Apple ourmia-like virus 3^f^	4e-94	37.7%	71%	53,219	2,123
Heterobasidion ambi-like virus 1 (HetAlV1)	RNA1	MZ502384	4R1	4,857	49.1	Armillaria mellea ambi-like virus 2^g^	3e-45	30.1%	34%	57,938	1,168
Heterobasidion ambi-like virus 2 (HetAlV2)	RNA1	MZ502385	RKU3.1.25	4,251	49.0	Armillaria borealis ambi-like virus 2^g^	7e-51	28.6%	41%	250,660	5,732
Heterobasidion ambi-like virus 3 (HetAlV3)	RNA1	MZ502386	RKU3.1.25	4,908	48.9	Armillaria ectypa ambi-like virus 1^g^	2e-78	33.2%	35%	535	11
Heterobasidion ambi-like virus 4 (HetAlV4)	RNA1	MZ502387	RKU3.1.25	4,913	48.7	Armillaria luteobubalina ambi-like virus 1^g^	4e-77	33.6%	34%	7,657	154
Heterobasidion partitivirus 21 (HetPV21)	dsRNA1	MZ502388	RKU3.2.124	1,928^h^	45.8^h^	Medicago sativa alphapartitivirus 2^i^	0.0	61.8%	91%	3,973	222
	dsRNA2	MZ502389	RKU3.2.124	1,767^h^	50.2^h^	Rosellinia necatrix partitivirus 13^j^	2e-122	45.1%	81%	4,797	268

*^*a*^The sequence having the highest alignment score (Max score) based on BlastX with nr database.*

*^*b*^Raw reads were mapped against the virus sequence using Geneious for RNA Seq assembler with custom sensitivity with 0 as a maximum mismatch % per read and not allowing gaps.*

*^*c*^Mean value generated by Geneious 10.2.6.*

*^*d*^Chiapello et al. (2020).*

*^*e*^Chiapello et al. (2021).*

*^*f*^Wright et al. (2020).*

*^*g*^Linnakoski et al. (2021).*

*^*h*^Without poly(A) tail.*

*^*i*^Bejerman et al. (2019).*

*^*j*^Telengech et al. (2020).*

**TABLE 2:** Presence/absence of virus strains and variants in the *H. parviporum* isolates included in the RNA-Seq library.

**Strain**	**HetNlV1^a^**	**HetOlV1^b^**	**HetAlV1**	**HetAlV2^c^**	**HetAlV3^d^**	**HetAlV4**	**HetRV6^e^**	**HetMV3^f^**	**HetPV2**	**HetPV4**	**HetPV13**	**HetPV16**	**HetPV21**
RKU3.1.25	–	–	–	pa1-a^g^	pa1-a^g^	pa1^g^	pa11-b	–	–	–	–	–	–
RKU3.2.124	–	pa1-a^g^	–	–	–	–	pa10-b	–	–	–	–	–	pa1^g^
RKU3.3.34	–	pa1-b	–	–	–	–	pa33	–	–	–	–	–	–
RKU172	pa1^g^	pa2	–	–	(pa1-b)	–	pa34	pa4	–	–	–	–	–
RT3.45	–	pa1-c	–	–	–	–	pa11-a	–	–	–	–	–	–
3R4	pa2	pa3	–	pa1-b	pa1-c	–	pa35-a	pa1-4	–	–	–	–	–
4R1	–	pa4	pa1-a^g^	–	(pa2-a)	–	pa35-b	(pa5)	–	–	–	–	–
LAP3.3.2*5	–	–	–	pa1-c	–	–	[pa32]	–	[pa1]	pa1	an1	[an1]	–

*^*a*^96.9% nt level identity.*

*^*b*^Strains 96.3–97.1% nt level identity, variants ≥99.5% nt level identity.*

*^*c*^Variants ≥98.1% nt level identity.*

*^*d*^Strains 97.1–97.5% nt level identity, variants ≥99.0% nt level identity.*

*^*e*^Strains 96.0–97.3% nt level identity, variants ≥99.7% nt level identity.*

*^*f*^Strains 93.7–94.6% nt level identity, variants ≥98.7% nt level identity.*

*^*g*^The virus strain deposited to GenBank. The abbreviation indicates the viral species (e.g., HetNlV1), while suffixes indicate strains (e.g., pa1, pa11), followed by sequence variants (a, b, c or 1, 2, 3 etc.). Brackets represent virus strains/variant not found present in the RNA-Seq library (probably due to inadequate read depth); square brackets represent virus strains confirmed to be absent from the host.*

*pa = virus strain/variant originating from *H. parviporum* isolate; an = virus strains originating from *H. annosum* isolate.*

The hosts of mycoviruses were detected with RT-PCR, and DNA of virus positive *H. parviporum* isolates was used to confirm the novel viruses were not integrated to the genome of the host.

### A Novel Bisegmented Narna-Like Virus Hosted by *Heterobasidion*

**FIGURE 2:**
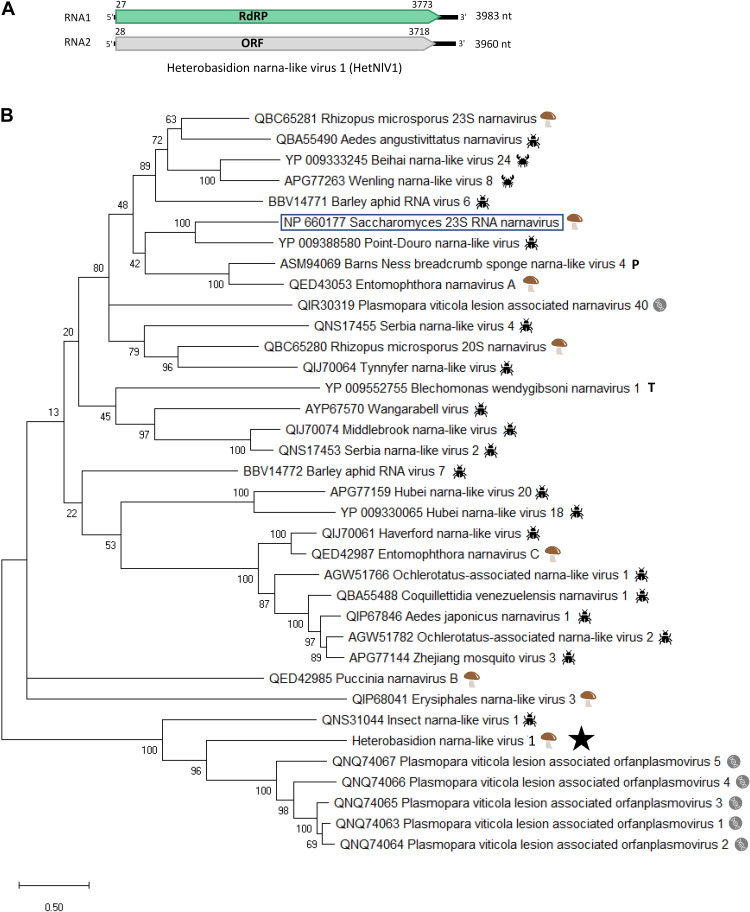
Genome organization and phylogenetic analysis of Heterobasidion narna-like virus 1 (HetNlV1). **(A)** Schematic presentation of HetNlV1 genome with UTRs and predicted ORFs with translation initiation and termination sites. **(B)** Phylogenetic tree including HetNlV1 and similar viruses based on an alignment of aa sequences of the RdRP encoding ORF generated using MAFFT v7.450 with Blosum45 substitution matrix. The viruses were selected based on BlastX similarity for HetNlV1 and Saccharomyces 23S RNA narnavirus (shown boxed). Evolutionary analyses were conducted in MEGA X (Kumar et al., 2018) with 500 bootstrap repeats (the percentage of trees in which the associated taxa clustered together is shown next to the branches). The evolutionary history was inferred by using the Maximum Likelihood method and Le_Gascuel_2008 model (Le and Gascuel, 2008). A discrete Gamma distribution was used to model evolutionary rate differences among sites (four categories +G +I). A star denotes the virus strain from *Heterobasidion parviporum*. The other symbols indicate the hosts: 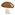 = fungi; 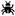 = insect; 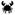 = crustacean; 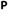 = porifera; 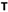 = trypanosomatida; 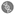 = metagenomic from Plasmopara viticola-caused leaf samples.

A Trinity contig (DN23600) of 7Het library showed the highest similarity (*e*-value 7e-74, percent identity 30.1%, query cover 72%) with the RdRP of Plasmopara viticola lesion associated orfanplasmovirus 1 (Chiapello et al., 2020), a narna-like virus. The contig had a mean coverage of 4,301 reads and was found to be hosted by two isolates, RKU172 and 3R4. As a second genome segment has been recently described in related viruses from *Sclerotinia sclerotiorum* (Jia et al., 2021), we carried out a BlastX analysis using the Sclerotinia sclerotiorum narnavirus 3 and 4 RNA2 sequences, which allowed us to identify a putative RNA2 segment. The genome of the putative narna-like virus named Heterobasidion narna-like virus 1 (HetNlV1) was Sanger sequenced using adapter-primed cDNA of isolate RKU172. The sequence of the complete RNA1 segment (3,983 nt) has one ORF encoding for a putative RdRP of 1,248 aa (predicted *M*_r_ = 140.491 kDa) with no conserved domains identified based on the NCBI conserved domain search but sharing similarity with predicted proteins of narna-like viruses (Table 1 and Figure 2A). The 3,960 nt long sequence of RNA2 includes Sanger confirmed 5′ UTR and CDS and one complete ORF encoding a predicted protein of 1,228 aa (predicted *M*_r_ = 134.058 kDa). Based on BlastX, the insect narna-like virus 1 (Chiapello et al., 2021) hosted by thrips (insect pest *Frankliniella occidentalis*) showed highest similarity with RNA2 of HetNlV1 (Table 1). Phylogenetic analysis showed placement of HetNlV1 in a highly supported cluster including viruses from *Plasmopara viticola* lesions and thrips (Chiapello et al., 2020, 2021; Figure 2B).

The partial, Sanger confirmed RNA1 (1,932 nt) hosted by 3R4 (Table 2) shared 96.9% nt level identity with the virus strain harbored by RKU172. The suitability of RNA-Seq for detecting virus strains was investigated by mapping raw reads against HetNlV1 using loose mapping criteria (allowing 20% mismatches per read). The Sanger confirmed region of the two HeNlV1 sequences included 58 SNPs of which all except one were also detected with variant calling (Supplementary Table 2).

### A Putative Member of *Botourmiaviridae* Hosted by Multiple *H. parviporum* Isolates

**FIGURE 3:**
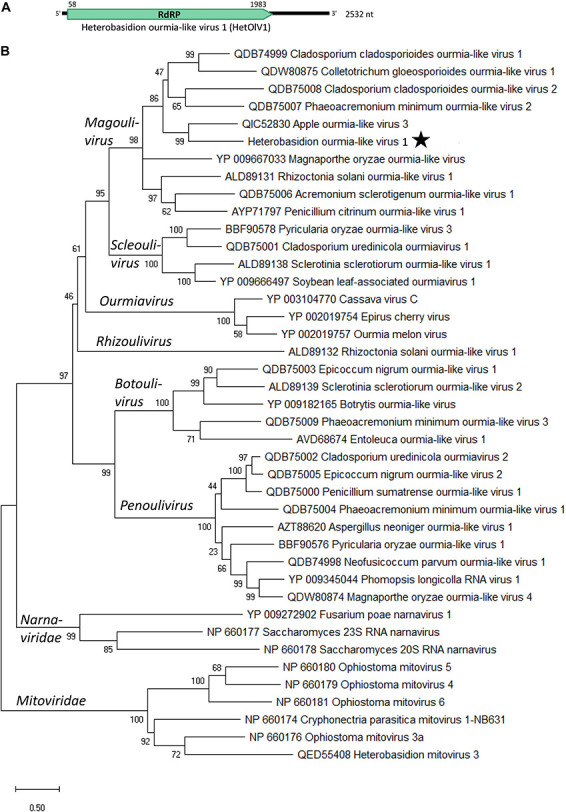
Genome organization and phylogenetic analysis of Heterobasidion ourmia-like virus 1 (HetOlV1). **(A)** Schematic presentation of HetOlV1 genome with UTRs and predicted ORF with translation initiation and termination site. **(B)** Phylogenetic tree including members of family *Botourmiaviridae* and exemplar isolates of *Mitoviridae* and *Narnaviridae* based on an alignment of aa sequences of the RdRP encoding ORF generated using MAFFT v7.450 with Blosum62 substitution matrix. Evolutionary analyses were conducted in MEGA X (Kumar et al., 2018) with 500 bootstrap repeats (the percentage of trees in which the associated taxa clustered together is shown next to the branches). The evolutionary history was inferred by using the Maximum Likelihood method and Le_Gascuel_2008 model (Le and Gascuel, 2008). A discrete Gamma distribution was used to model evolutionary rate differences among sites (four categories +G +I). A star denotes the virus strain from *Heterobasidion parviporum*.

A Trinity contig (DN24714) showing similarity with ourmia-like viruses and having the mean coverage of more than 3,640 reads (with loose mapping criteria) was detected by RT-PCR in six *H. parviporum* isolates in the RNA-Seq library (Table 2). The sequence of this virus hosted by isolate RKU3.2.124 and named Heterobasidion ourmia-like virus 1 (HetOlV1) was confirmed with Sanger sequencing, and complete 5′ and partial 3′ UTRs were obtained using adapter ligated cDNA (Table 1 and Figure 3A). The Sanger confirmed genome of 2,532 showed highest similarity with the RdRP of apple ourmia-like virus 3 (Wright et al., 2020; Table 1). The most closely related classified ourmiavirus is Cladosporium cladosporioides ourmia-like virus 1 (Nerva et al., 2019), a magoulivirus, with ca. 36% BlastX identity (65% query cover). The single ORF present in the genome encodes for a 641 aa long putative RdRP (predicted *M*_r_ = 72.491 kDa). No conserved domains were detected based on the NCBI conserved domain search, but the putative RdRP shared conserved amino acids with other RdRPs of ourmia-like viruses. Based on phylogenetic analysis, HetOlV1 is included in a highly supported cluster with classified members of genus *Magoulivirus* (Figure 3B).

The ∼970 nt long Sanger confirmed sequence of HetOlV1 hosted by isolates RKU3.2.124, RKU3.3.34, and RT3.45 showed minor sequence variations sharing more than 99.4% nt level sequence identities, while the identity of sequences from 3R4, 4R1, and RKU172 varied between 96.3 and 97.1% (Table 2). Therefore, we deemed that there were four different strains of HetOlV1 in the *H. parviporum* isolates included in the RNA-Seq library, one of them represented by three different variants. The studied sequences included altogether 58 SNP sites, all of which were detected by variant calling of mapped reads (Supplementary Table 2).

### Four Ambi-Like Viruses

**FIGURE 4:**
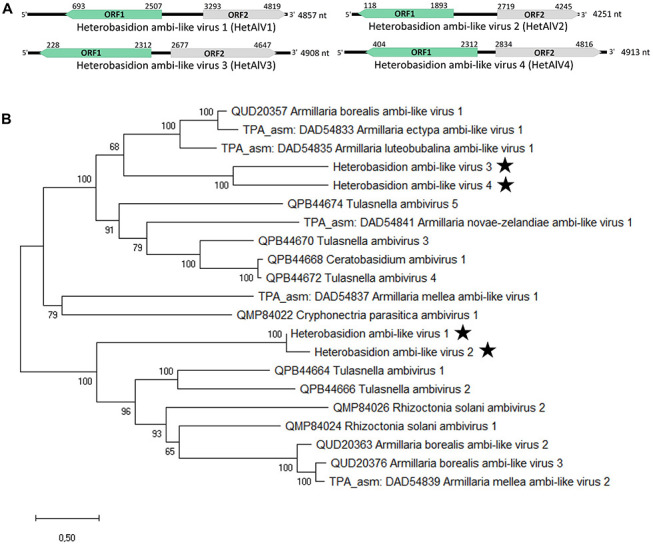
Genome organization and phylogenetic analysis of Heterobasidion ambi-like viruses 1–4 (HetAlV1–4). **(A)** Schematic presentation of HetAlV1–4 genomes with UTRs and predicted ORFs with translation initiation and termination sites. Green-colored ORFs represent the putative RdRPs. **(B)** Phylogenetic tree of the ambi-virus like viruses. The predicted aa sequences of putative RdRPs were aligned in MEGA X (Kumar et al., 2018) with MUSCLE and tree was constructed with the LG (+F) model with G+I rates among sites (best-fit model according to ModelFinder) and 500 bootstrap repetitions. All positions with less than 95% site coverage were eliminated, i.e., fewer than 5% alignment gaps, missing data, and ambiguous bases were allowed at any position. The Heterobasidion ambi-like viruses are designated with a star.

The 7Het library contained Trinity contigs of four distinct virus genomes encoding proteins having similarity with the recently discovered “ambiviruses” (Sutela et al., 2020; Forgia et al., 2021). Typically, the *de novo* assembled contigs of ambiviruses show variation in the orientation of ORFs including presence of partial or complete dimers of the monomeric genome. In the 7Het library, two of the putative ambi-like viruses were represented by near full-length Trinity contigs. In the case of two other ambi-like viruses, several partial Trinity contigs were found, aligned, and used for read mapping and construction of a final consensus sequence. The near complete genomes showed highest similarity with Armillaria ambi-like viruses (Linnakoski et al., 2021) and were named as Heterobasidion ambi-like virus 1–4 (HetAlV1–4, Table 1). HetAlV1 and HetAlV4 were hosted by one *H. parviporum* isolate (4R1 and RKU3.1.25, respectively), whereas HetAlV2 and HetAlV3 had multiple hosts (Table 2). In the case of HetAlV2 and HetAlV3, the sequence covering both ORFs was Sanger confirmed from isolate RKU3.1.25, which hosted three of the ambi-like viruses (HetAlV2–4; Table 1). No conserved domains were detected for any of the ambi-like proteins, but the longer of the predicted proteins contained the GDD motif and was thus considered as the putative RdRP (Figure 4A). The predicted Mr values of putative RdRPs were 69.023, 67.962, 80.374, and 80.709 kDa for HetAlV1 hosted by 4R1 and for HetAlV2–4 hosted by RKU3.1.25, respectively. The HetAlV1 and HetAlV2 sequences shared 79.8% nt sequence identities and 83.1% sequence identities in the aa of putative RdRP. Both HetAlV1 and HetAlV2 had less than 36% sequence identities with HetAlV3 and HetAlV4 at nt and aa (putative RdRP) level. The nt and aa level identities of HetAlV3 and HetAlV4 were 33.8 and 39.9%, respectively. Based on small scale phylogenetic analysis of the ambi-like viruses harbored by altogether six different fungal genera, the *H. parviporum* viruses were placed into two different clusters (Figure 4B).

Screening with RT-PCR revealed that HetAlV2 was hosted by three and HetAlV3 by four *H. parviporum* isolates (Table 2). The partial Sanger confirmed HetAlV2 sequences (∼930 nt) hosted by RKU3.1.25, 3R4 and LAP3.3.2^∗^5 contained altogether 24 SNPs and shared more than 98% sequence identities. Thus, the three HetAlV2 viruses were considered as variants of the same strain and designed as HetAlV2-pa1-a, pa1-b, and pa1-c, respectively (Table 2). All the SNPs identified aligning the Sanger confirmed HetAlV2 sequences were also detected with variant calling (Supplementary Table 2). The HetAlV3 sequences of RKU3.1.25, RKU172, and 3R4 shared more than 98.9% sequence identities and were designated as HetAlV3-pa1-a, pa1-b, and pa1-c, respectively. The HetAlV3 of 4R1 showed lower similarity (97.1–97.5%) and was considered as a second strain of HetAlV3 (HetAlV3-pa2; Table 2). The alignment of ∼630 nt long Sanger confirmed HetAlV3 sequences contained 23 SNPs of which variant calling found 19 (Supplementary Table 2). Moreover, the Sanger confirmed sequences of HetAlV3-pa1-b, pa1-c, and pa2 were not covered by the raw reads pointing to inadequate read-depth. Also the coverage of HetAlV3-pa1-a was low, only 535 raw reads were mapped against the near-full length sequence when no mismatches were approved (Table 1). However, the HetAlV3 amplicons could not be produced using total nucleic acids of three different hosts as a template, thus indicating HetAlV3 was not integrated to the genome of the hosts.

### Members of *Mitoviridae*, *Curvulaviridae*, and *Partitiviridae*: New Strains and Variants and Differences in Read Coverage

#### *Mitoviridae*: Heterobasidion Mitovirus 3

Three variants of Heterobasidion mitovirus 3 strain pa1 (HetMV3-pa1) have been described at the Ruotsinkylä research forest (plot 7R) earlier (Vainio, 2019). These variants were detected in two clones and nine *Heterobasidion* isolates. In this study, fungal isolates 3R4, 4R1, and RKU172 originating from three more plots at the Ruotsinkylä research forest were found to be infected with HetMV3 based on screening with RT-PCR. The Sanger confirmed HetMV3 sequence hosted by 3R4 was very closely related to HetMV3-pa1 variants 1–3 previously found from the 7R plot (Vainio, 2019), sharing >98.6 nt identities with them, whereas the HetMV3 sequences originating from 4R1 and RKU172 showed identities of less than 95% (Table 2). Therefore, the virus hosted by 3R4 was considered as the fourth variant of HetMV3-pa1 (HetMV3-pa1-4), whereas the viruses in RKU172 and 4R1 were considered different HetMV3 strains, designated as HetMV3-pa4 and pa5 (Table 2).

Interestingly, read mapping revealed a very high read depth for HetMV3-pa1-4 and HetMV3-pa4 but the presence of HetMV3-pa5 could not be confirmed. Thus, ca. 0.38 and 0.21 million reads mapped to 1,452 nt long Sanger confirmed sequences of HetMV3-pa1-4 and pa4, respectively, when no mismatches were allowed in the mapping. In contrast, the HetMV3-pa5 was only partially covered, and moreover, less than 15 raw reads were aligned to the sites containing unique SNPs of the strain. For the variant calling, mapping was conducted with loose parameters and all but one SNPs of HetMV3-pa1-4 and pa4 were detected (Supplementary Table 2), but the unique SNPs of HetMV3-pa5 were not.

#### *Curvulaviridae*: Heterobasidion RNA Virus 6

Eight different strains of the orthocurvulavirus Heterobasidion RNA virus 6 (HetRV6) had been detected at the Ruotsinkylä research forest (plot 7R) earlier (Vainio et al., 2015a). In this study, seven *H. parviporum* isolates included in the RNA-Seq analysis were infected with HetRV6 based on RT-PCR (Table 2). The HetRV6-pa11 hosted by RT3.45 (Vainio et al., 2012) shared 99.7% sequence identity with the HetRV6 hosted by RKU3.1.25. Hence, this HetRV6 variant was designated as HetRV6-pa11-b. The HetRV6 in RKU3.2.124 showed high similarity (99.8%) with HetRV6-pa10 (Vainio et al., 2012) and was named HetRV6-pa10-b. The HetRV6 in RKU3.3.34 and RKU172 were considered as new strains of HetRV6 (HetRV6-pa33 and pa34, respectively) based on sequence identities ranging between 96.0 and 97.2%. The HetRV6 sequences of 3R4 and 4R1 shared 99.9% nt level identity and had 96–97% identities with other HetRV6 sequences at the study plots and were thus considered as variants of a new HetRV6 strain (HetRV6-pa35-a and pa35-b, respectively; Table 2).

A total of 10,635 reads mapped to dsRNA1 of HetRV6-pa33 and all 138 SNP identified in Sanger confirmed complete ORFs of the seven HetRV6 strains and variants listed above were detected by variant calling (Supplementary Table 2). Using corresponding parameters in mapping and variant calling, a total of 158 SNPs could be identified in the dsRNA2 of HetRV6. In this analysis, the reads were mapped against the unpublished HetRV6-ab6 sequence available in GenBank (MK468678), which resulted in 15,377 mapped reads against the 1859 nt sequence. Notably, the number of reads per infected host isolate was considerably lower for HetRV6 (a bisegmented dsRNA virus) than for mitoviruses (non-segmented positive-sense RNA viruses).

#### *Partitiviridae*: Three Species of Genus *Alphapartitivirus*

The three partitiviruses detected in the RNA-Seq library were the alphapartitiviruses HetPV4-pa1 and HetPV13-an1 known to be present in the LAP3.3.2^∗^5 multivirus isolate (Hantula et al., 2020), and one new partitivirus newly detected in isolate RKU3.2.124 (Table 2).

**FIGURE 5:**
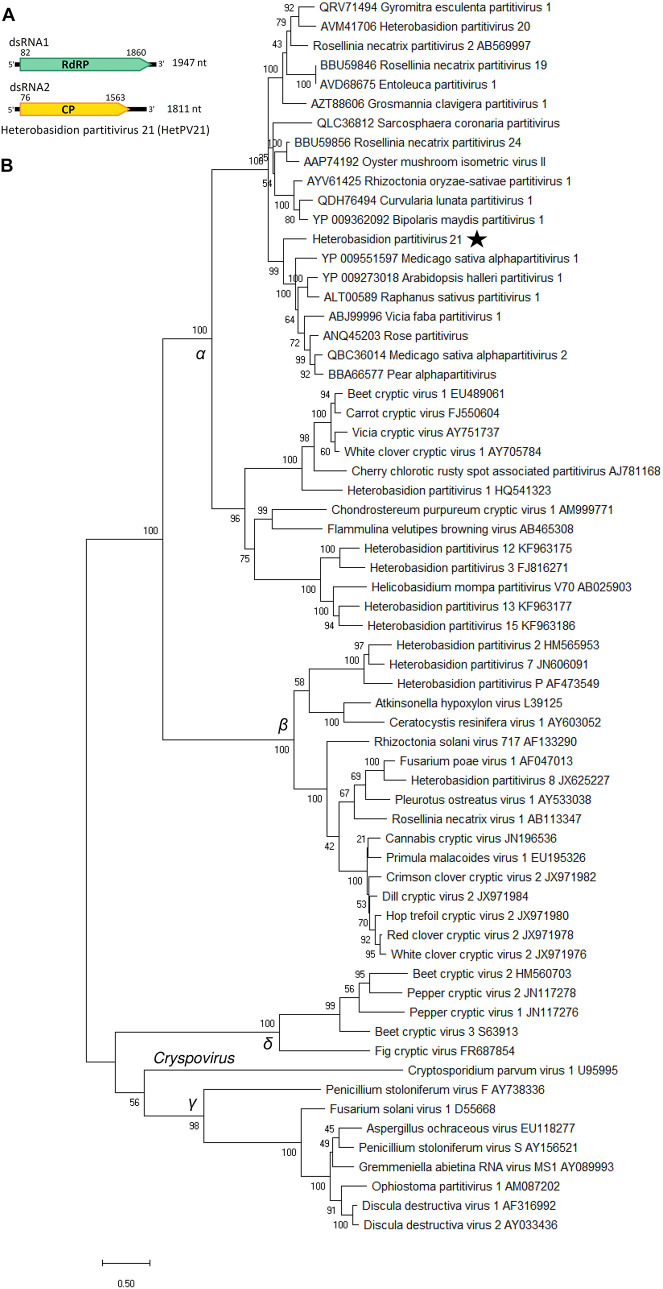
Genome organization of Heterobasidion partitivirus 21 (HetPV21) and phylogenetic analysis of partiti- and related virus strains. **(A)** Schematic presentation of HetPV21 genome with UTRs and predicted ORFs with translation initiation and termination sites. **(B)** Phylogenetic tree including members of family *Partitiviridae* (accession number after virus name) and related unclassified virus strains (accession number before virus name) based on an alignment of aa sequences of the RdRP encoding ORF generated using MAFFT v7.450 with Blosum62 substitution matrix. Evolutionary analyses were conducted in MEGA X (Kumar et al., 2018) with 500 bootstrap repeats (the percentage of trees in which the associated taxa clustered together is shown next to the branches). The evolutionary history was inferred by using the Maximum Likelihood method and Le_Gascuel_2008 model (Le and Gascuel, 2008). A discrete Gamma distribution was used to model evolutionary rate differences among sites (four categories +G +I). A star denotes the virus strain from *Heterobasidion parviporum*, and Greek symbols indicate the partitivirus genera (α = *Alphapartitivirus*, β = *Betapartitivirus*, γ = *Gammapartitivirus*, δ = *Deltapartitivirus*).

The new partitivirus hosted by isolate RKU3.2.124 was named Heterobasidion partitivirus 21 (HetPV21), and its complete genome sequence with 5′ and 3′ UTRs was determined (Table 1 and Figure 5A). The dsRNA1 of HetPV21 is most similar to Medicago sativa alphapartitivirus 2 (Bejerman et al., 2019) and the sequence contains single ORF encoding for a putative RdRP of 592 aa (predicted *M*_r_ = 69.022 kDa). The dsRNA2 of HetPV21 encoding a putative CP (495 aa, predicted *M*_r_ = 54.245 kDa) shows highest similarity to Rosellinia necatrix partitivirus 13 (Telengech et al., 2020; Table 1). This level of sequence identity fulfills the criteria of a new species in family *Partitiviridae*, genus *Alphapartitivirus* (Vainio et al., 2018b). The first 14 nucleotides of the two genome segments were identical. Read mapping revealed mean sequence coverage of >220 reads mapped to HetPV21 dsRNA1 and dsRNA2 (Table 1). Interestingly, phylogenetic analyses revealed that HetPV21 is included in a highly supported virus cluster including only plant alphapartitiviruses (Figure 5B).

We also completed the genome sequence of HetPV4-pa1 by determining its dsRNA2 sequence from its original host isolate RT3.49C from plot 4R as described earlier for dsRNA1 (Vainio et al., 2011). The two genome segments shared a common conservative 5′ sequence of six nucleotides. dsRNA2 encoded a putative CP of 483 aa, which was most closely related to the Rosellinia necatrix partitivirus 9 (97% query cover, 32.23% BLASTP identity; Arjona-Lopez et al., 2018). The length of dsRNA2 was 1718 nt excluding an interrupted poly(A) tail of ca. 101 nt (the exact length and number of interrupting T nucleotides may vary). Read mapping revealed mean sequence coverage of 111 (1,881 reads) mapped to HetPV4-pa1 dsRNA2 and 515 (10,300 reads) for dsRNA1 when the poly(A) tails were excluded.

The read coverage was also determined for HetPV13-an1, which has been completely sequenced earlier (Kashif et al., 2015). The read coverage in the multivirus isolate was found to be very low (only 629 reads mapped to dsRNA1 with a mean read depth of 33), and hence, special care should be given to determination of sufficient sequence coverage in the HTS in order to obtain accurate detection of partitiviruses.

#### Occurrence and Stability of Virus Co-infections

The *H. parviporum* isolates examined by RNA-Seq were infected altogether by 11 different viruses, of which 6 infected more than one isolate (Table 2). The infections of *H. parviporum* isolates were confirmed with RT-PCR, and the amplicons were Sanger sequenced which enabled us to distinguish the unique virus strains hosted by specific fungal isolates. All fungal isolates had mixed virus infections ranging from two to six infections. HetRV6, HetOlV1, and HetAlV3 infections were most prevalent, whereas HetAlV1 and HetAlV4 as well as most of the partitiviruses were hosted by one isolate only (Table 2).

The RNA-Seq library included *H. parviporum* strain LAP3.3.2^∗^5 infected with the orthocurvulavirus HetRV6–32 in addition to four partitiviruses, which had been introduced to the fungal strain by laboratory pairing experiments (Hantula et al., 2020). Based on the inspection of the RNA-Seq library by contig assembly, read mapping and variant calling (see above) as well as RT-PCRs (conducted also from cDNA originating from re-grown LAP3.3.2^∗^5), the artificially infected fungal strain had been cured of two of the four partitivirus infections (the betapartitivirus HetPV2 and alphapartitivirus HetPV16) and HetRV6 since it was generated in 2013 (Hantula et al., 2020; Table 2). The isolate still harbored the alphapartitiviruses HetPV4 and HetPV13 (Table 2), while the absence of HetPV2 and HetPV16 was evident as no reads mapped to the genome sequences of these viruses. In the case of HetRV6-pa32, read mapping could not confirm the presence of this particular strain as raw reads were not aligned to the sequence containing unique SNPs. Thus, the RNA-Seq analysis revealed that virus co-infections generated by laboratory transmission experiments were not stable in the case of LAP3.3.2^∗^5.

### Occurrence of the Novel Mycoviruses in *H. parviporum* Clones at a Boreal Forest Site

The presence of HetNlV1, HetOlV1, and HetAlV1–4 was studied by RT-PCR in selected *H. parviporum* isolates (*N* = 43) collected in 2005 and 2012 from the 7R study plot (Vainio et al., 2015a). The examined isolates represented nine clonally spreading *H. parviporum* individuals, three of which were homokaryotic (sampling points: 6, 14, and 117-05; Figure 1 and Table 3) and revealed to be commonly infected by ourmia- and ambi-like viruses, whereas narna-like viruses similar to HetNlV1 were not detected (Figure 1 and Table 3). The clonal structure as well as the presence of orthocurvulaviruses (HetRV6) and mitoviruses had been investigated already earlier at the same forest plot, and also partitiviruses had been screened earlier by cellulose chromatography followed by reverse transcription of viral RNA and cloning (Vainio et al., 2015a; Vainio, 2019). The results of these previous studies are summarized together with our new data in Table 3 to provide the complete virus infection status of the analyzed *H. parviporum* isolates.

**TABLE 3:** Summary of the virus infection status of *H. parviporum* strains and virus variants and strains of 7R field site.

**Clone size^a^**	**Sampling point**	**Substrate**	**Current study**					**Previously published data**			
			**HetOlV1** ^b^	**HetAlV1** ^b^	**HetAlV2** ^b^	**HetAlV3** ^b^	**HetAlV4** ^b^	**HetPV2** ^c^	**HetPV7** ^c^	**HetPV9** ^c^	**HetRV6** ^c^
3	95^d^	T	pa5-a/pa5-a	−/−	−/pa1-d	pa2-b/pa2-b	−/−	+/+	−/−	−/−	−/+
	96^d^	T	pa5-a/NA	−/NA	−/NA	pa2-b/NA	−/NA	+/+	−/−	−/−	−/−
	117^d,e,f^	S	NA/pa5-b	NA/−	NA/pa1-d	NA/−	NA/−	NA/+	NA/−	NA/−	NA/+
5	195^d^	S	−/pa5-c	−/pa1-b	−/pa1-d	−/pa1-d	−/−	−/−	+/+	−/−	+/+
	218^d^	T	pa5-c/pa5-c	pa1-b/pa1-b	pa1-d/pa1-d	pa1-d/pa1-d	−/−	−/−	+/+	−/−	−/−
	226^d,e^	T	pa5-c/pa5-d	pa1-b/pa1-b	−/pa1-d	pa1-d/pa1-d	−/−	−/−	+/+	−/−	+/−
	241^d^	S	pa5-d/NA	pa1-b/NA	–/NA	pa1-d/NA	−/NA	−/NA	+/NA	−/NA	+/NA
	274^d^	S	pa5-c/−	pa1-b/pa1-b	pa1-d/−	−/pa1-d	−/−	−/−	+/+	−/−	+/+
7	2^d^	T	pa5-e/ND	−/ND	−/ND	pa2-b/ND	−/ND	−/+	−/−	−/−	−/−
	4^d^	T	pa5-f/−	−/−	−/−	pa2-b/-	−/−	−/−	−/−	−/−	−/−
	5^d,g^	T	pa5-g/pa5-g	−/−	−/−	−/pa2-b	−/−	+/−	−/−	−/−	+/+
	10^d^	T	pa5-f/pa5-f	−/−	−/−	pa2-b/pa2-b	−/−	−/−	−/−	−/−	−/−
	12^d^	T	pa5-e/pa5-e	−/−	−/−	−/pa2-b	−/−	+/+	−/−	−/−	−/+
	16^d^	T	−/pa5-c	−/−	−/pa1-d	−/−	−/−	−/−	−/−	−/−	−/−
	18^d^	T	pa5-c/pa5-c	−/−	pa1-d/pa1-d	pa2-b/pa2-b	−/−	+/+	−/−	−/−	−/−
5	82^d,h^	S	−/−	pa1-c/pa1-c	−/−	−/pa1-e	−/−	−/−	−/−	−/−	+/+
	83^d,g,i^	S	−/−	−/pa1-c	−/−	−/pa1-e	−/−	−/−	−/−	−/−	−/+
	112^d,e,g^	T	−/−	−/pa1-c	−/−	−/pa1-e	−/−	−/−	−/−	−/−	−/−
	123^d,h^	S	−/−	−/pa1-c	−/−	−/pa1-e	−/−	−/−	−/−	−/−	+/+
	124^d,h^	S	−/−	pa1-c/pa1-c	−/−	−/−	−/−	−/−	−/−	−/−	+/+
2	227^d^	T	pa6/NA	pa1-b/NA	pa1-d/NA	pa1-d/NA	−/NA	−/NA	+/NA	+/NA	+/NA
	242^d^	S	pa6/NA	pa1-b/NA	pa1-d/NA	pa1-d/NA	pa2/NA	−/NA	+/NA	+/NA	+/NA
1	6^d^	T	−/NA	−/NA	−/NA	−/NA	−/NA	−/NA	−/NA	−/NA	−/NA
1	14^d^	T	−/−	−/−	−/−	−/pa2-b	−/−	−/−	−/−	−/−	−/−
1	111^e^	T	ND/−	ND/pa1-c	ND/−	ND/−	ND/−	−/−	−/−	−/−	−/−
1	117^j^	S	−/NA	−/NA	−/NA	pa2-b/−	−/NA	+/NA	−/NA	−/NA	−/NA

*^*a*^The number of isolates of a clone analyzed in the present study.*

*^*b*^The presence of virus infection and virus strains and variants (see Table 2 for the utilized thresholds) determined from *H. parviporum* strain isolated in year 2005/2012.*

*^*c*^The presence of virus infection according to Vainio et al. (2015a) in strains isolated in year 2005/2012.*

*^*d*^Both 2005 and 2012 strains were HetNlV1 negative.*

*^*e*^*H. parviporum* strain isolated 2012 was HetMV3 negative (Vainio, 2019).*

*^*f*^Only isolate from year 2012 represented same clone as 95 and 96.*

*^*g*^*H. parviporum* strain isolated 2005 was HetMV3 negative (Vainio, 2019).*

*^*h*^*H. parviporum* strains isolated 2005 and 2012 were HetMV3 positive (Vainio, 2019).*

*^*i*^*H. parviporum* strain isolated 2012 was HetMV3 positive (Vainio, 2019).*

*^*j*^Only 2005 isolate presented unique clone.*

*T = sampling source a standing tree; S = sampling source a stump; − = not infected; + = infected; NA = not available: *H. parviporum* strain representing the same clone not found in the sampling point of 7R; ND = not determined: sampling point not included to the screening.*

HetOlV1 was found in 2005 in four heterokaryotic clones, which also contained isolates free of HetOlV1 infection (Figure 1A; Supplementary Figure 1; and Table 3). The HetOlV1 infections persisted during the 7-year sampling period in all sampling points (including both stumps and trees as an isolation source; see Vainio et al., 2015a and Figure 1A) examined, with the exception of two (4-12 and 274-12). In 2012, HetOlV1 was found in isolates from three new sampling points (16-12, 117-12, and 195-12; Figure 1B; Supplementary Figure 1; and Table 3). Two HetOlV1 strains occurred at the site, HetOlV1-pa5 and pa6 (nt identities 96.9–97.6%), of which HetOlV1-pa5 infected isolates of three clones. Minor sequence polymorphism (up to two SNPs) was detected in the HetOlV1-pa5 sequences between and within clones and also between strains of sampling point 226 isolated in 2005 and 2012 (Supplementary Figure 1 and Table 3). Moreover, identical HetOlV1 variants were not found present in more than one host clone.

All *H. parviporum* ambi-like viruses (HetAlV1–4) were confirmed to occur at 7R study plot in fungal strains isolated in 2005 (Figure 1 and Table 3); HetAlV1 and –2 infected three clones, HetAlV3 five (including a homokaryotic clone from sampling point 117) and HetAlV4 one. Two HetAlV1-pa1 variants, one HetAlV2-pa1 variant, and one variant of both HetAlV3-pa1 and –pa2 occurred at the site 2005 and a second HetAlV3-pa1 variant was detected at a new sampling point in 2012 (Figure 1 and Table 3). The fungal isolates of a certain clone were always found to be infected with the same specific virus variant. The nt level sequence identities between variants of single virus were high (>97% nt level sequence identities for HetAlV1/HetAlV2/HetAlV3). Variants of HetAlV1–3 were confirmed to occur at the study site for 7 years in fungal strains isolated from both stumps and trees, and five new HetAlV1 and –2 infections as well as nine HetAlV3 infections were detected in new sampling points after 7-year study period. Furthermore, at sampling points 4-12 and 274-12, infections of ambi-like viruses were lost (in addition to HetOlV1 infection). Of the ambi-like viruses, HetAlV3 infections were the most common as altogether 26 isolates were HetAlV3 positive of which two represented homokaryotic isolates. During the 7-year sampling period, HetAlV3-pa1-d and pa2-b detected at the study site in 2005 remained mostly stably and a third HetAlV3 variant was found as four isolates of a single clone were newly infected with HetAlV3-pa1-e in 2012 (Figure 1B and Table 3). In contrast to HetAlV1–3, HetAlV4 was only detected in one *H. parviporum* isolate (242-05). The sequence identity of HetAlV4-pa2 was 95.6% with the HetAlV4-pa1 hosted by RKU3.1.25 included in the RNA-Seq. This particular clone was found in the forest plot only in 2005.

The novel ssRNA viruses discovered here, HetAlV3, HetOlV1, and HetAlV2, had infected 60, 56, and 42% of the 7R fungal isolates screened in this study, respectively. This presents a significant increase in the viral content of the isolates when compared to our earlier dsRNA-based analysis (Vainio et al., 2015a), where the orthocurvulavirus HetRV6 was found to be the most common virus with a prevalence of 43% among the isolates selected for the present study. Consequently, viral co-infections were also highly common; there were only six virus-free isolates and two isolates infected with one mycovirus only. Double and quadruple infections were the most frequent co-infections in the strains isolated 2005 and 2012, respectively, detected in seven isolates. Moreover, a single host isolate (242-05) harbored altogether eight viruses including a botourmiavirus, four ambi-like viruses, alphapartitiviruses, a betapartiti-, and an orthocurvulavirus (Table 3). Overall, the virus content of the isolates seemed to increase in time: the average number of infections was 2.4 and 3.1 for 2005 and 2012, respectively, when the virus status of strains isolated in both 2005 and 2012 from the same sampling point was included in the calculation.

## Discussion

The RNA-Seq approach used in the present study successfully revealed members of all three virus families known to infect *Heterobasidion* species: *Partitiviridae* (three species, one of which previously unknown), *Mitoviridae* (HetMV3 represented by two new strains and one variant), and *Curvulaviridae* (one known species; three new strains and three new variants) but also viruses representing groups not detected in isolates of *Heterobasidion* earlier: a putative narna-like virus, an ourmia-like virus, and four ambi-like viruses. Therefore, only half of the virus families or unclassified family level groups detected in this study had been discovered in *Heterobasidion* spp. prior to the employment of RNA-Seq, and only two of them had been discovered based on dsRNA screening. A similar situation has been revealed in several other fungal plant pathogens recently subjected to HTS analysis despite extensive screening for viruses based on traditional methods earlier (for instance Arjona-Lopez et al., 2018; Jia et al., 2021; Ruiz-Padilla et al., 2021).

We also compared virus isolate detection by RNA-Seq SNP calling to that based on Sanger sequencing, and found that mapping of RNA-Seq reads successfully revealed polymorphic nucleotide sites present in HetRV6, HetAlV2, HetNlV1, and HetOlV1. Moreover, based on the read mapping and variant calling, one of three Sanger confirmed HetMV3 and two of four HetAlV3 isolates were found to be absent from the RNA-Seq library. In the case of HetMV3, the read coverage was extremely high, and we can conclude that the absence of HetMV3-pa5 reads was most likely not due to low read depth but rather to sample processing prior to RNA-Seq (revival of isolates from cold storage, liquid cultivation and sample preparation). The raw read coverages of all HetAlV3 variants were very low and, thus, the sequencing coverage was not optimal for this particular virus. *Heterobasidion* partitiviruses are typically stable in +4°C for years or even decades when stored in adequate conditions, i.e., stable temperature and moisture (Kashif et al., 2015). Yet, the RNA-Seq and RT-PCRs revealed that infections created by laboratory pairing experiments can be unstable as a fungal strain with sextuple infection had lost two of the introduced partitiviruses.

The virus family *Narnaviridae* was recently divided into two families when mitoviruses were split into a new family *Mitoviridae* (ratified by the ICTV Executive Committee in 2019).^1^ Currently, there are only two classified species in family *Narnaviridae*. However, in addition to numerous typical narnaviruses, several new groups of narna-like viruses have been recently discovered in fungi and oomycetes. This includes bisegmented narnaviruses with a split polymerase palm domain (“splipalmiviruses”; Sutela et al., 2020) in *O. maius* and similar viruses with three or four genome segments in *Aspergillus fumigatus* and *Magnaporthe oryzae*, respectively (Chiba et al., 2021). In addition, *S. sclerotiorum* was shown to host similar narna-like viruses with both three and four segment genomes (Jia et al., 2021). Bisegmented “orfanplasmoviruses” (Chiapello et al., 2020) have a single segment coding for a putative RdRP and a second segment for a hypothetical protein with unknown function (Chiapello et al., 2020, 2021; Jia et al., 2021). Moreover, numerous narna-like viruses have been detected by HTS in arthropods (Shi et al., 2016) and by transcriptomic datasets from arthropods, fungi, and plants (Dinan et al., 2020). Interestingly, long reverse-frame ORFs were found to be common in one clade of narna-like viruses, which presents the first putative case of positive-sense viruses encoding a protein on the negative strand (Dinan et al., 2020). The narnavirus detected in this study, HetNlV1, represents a first “orfanplasmovirus” from a basidiomycete host, whereas the other viruses that formed a highly supported cluster with the HetNlV1 are from metagenomics studies of leaf samples infected by the oomycete *P. viticola* (Chiapello et al., 2020) or thrips (Chiapello et al., 2021).

Positive-sense RNA viruses resembling members of family *Botourmiaviridae* also occurred in the *H. parviporum* isolates. Fungal ourmia-like viruses are currently classified in five genera and have only one genome segment encoding the RdRP, whereas plant viruses in the genus *Ourmiavirus* have two more genome segments encoding a movement protein and a capsid protein (Turina et al., 2017; Ayllón et al., 2020). Based on phylogenetic analysis, the ourmia-like virus in *Heterobasidion* (HetOlV1) resembles viruses in the genus *Magoulivirus*. Based on ICTV criteria for the classification of botourmiaviruses (Ayllón et al., 2020), aa sequence identities of putative RdRP proteins between viruses belonging to different species of the genus *Magoulivirus* are less than 90%. Based on this species delimitation threshold and virus classification guidelines in the age of metagenomics (Simmonds et al., 2017), the coding complete genome sequence of HetOlV1 seems to represent a new virus species in genus *Magoulivirus*. A new taxonomical proposal submitted to the ICTV in 2021^2^ suggests the generation of six new genera and 109 new species in family *Botourmiaviridae*. In that proposal, genus *Magoulivirus* remains a single genus with 30 new species mostly from the oomycete *P. viticola* but also from *Botrytis cinerea* and *Macrophomina phaseolina*. The plant virus apple ourmia-like virus 3 (Wright et al., 2020) sharing the highest sequence similarity with the HetOlV1 is also proposed to be a new member of genus *Magoulivirus*.

“Ambiviruses” are bicistronic viruses with a ∼5 kb linear genome containing two ORFs in ambisense orientation and, which thus far, have been found by means of HTS in *Armillaria*, *Ceratobasidion*, *Cryphonectria*, *Rhizoctonia*, and *Tulasnella* spp. (Sutela et al., 2020; Forgia et al., 2021; Linnakoski et al., 2021). They are taxonomically unassigned and could, together with some members of the order *Bunyavirales*, formally qualify as a new class in the Baltimore classification or could be considered belonging in both class IV and V (Koonin et al., 2021). In the present study, four novel ambi-like viruses were harbored by *H. parviporum* isolates included in the RNA-Seq library and the same viruses were also found present in the isolates of the 7R forest site. One of the isolates included in the RNA-Seq analysis hosted three of the ambi-like viruses (HetAlV2–4), whereas an isolate of the 7R forest site was infected with all four ambi-like viruses (HetAlV1–4), supporting the assumption (based on the sequence identities) that all four represent distinct virus species, including HetAlV3 with low read depth in the RNA-Seq. Furthermore, PCR analysis of DNA templates as well as new infections by HetAlV3-pa1-e in a *Heterobasidion* clone at our study site during the 7-year sampling period indicates that HetAlV3 is not integrated to the host DNA.

As our RNA-Seq analysis suggested that a major part of viral diversity in *Heterobasidion* remains undetected by traditional methods, we investigated samples from an extensively studied forest plot for the occurrence of novel ssRNA viruses discovered in the present study. Previously known *Heterobasidion* viruses from the Ruotsinkylä site (two study plots) were the alphapartitiviruses HetPV4 and HetPV9, the betapartitiviruses HetPV2 and HetPV7, the orthocurvulavirus HetRV6 (eight distinct strains), and the mitovirus HetMV3 (Vainio et al., 2015a; Vainio, 2019). By examining the strains isolated from the study site 7R, we found that the botourmiavirus HetOlV1 and four ambi-like viruses HetAlV1–4 detected with HTS infected the *H. parviporum* clones. We found three cases where the same variant of an ambi-like virus had infected two adjacent clones (Figure 1 and Table 3), suggesting that these viruses are being transmitted between *H. parviporum* strains *via* hyphal contact. In addition, a novel HetAlV3 variant was detected in an aging *H. parviporum* clone during the 7-year study period, but the origin of the variant cannot be confirmed at this point (i.e., whether the infection is spore-mediated or obtained by hyphal contact with a clone outside the study area). During the 7-year study period, we also detected within-clone spread of HetOlV1 and HetAlV1–3. Moreover, we were able to confirm that HetOlV1 and HetAlV1–3 infections persisted at the study site during the study period of 7 years, indicating that infections by these ssRNA viruses are mostly stable. Their prevalence was also higher than that of partitiviruses and similar to that of orthocurvulaviruses at the site (Vainio et al., 2015a). In the case of ambi-like viruses, no spatial or temporal variation was observed in the virus variants within specific clones. In contrast, HetOlV1-pa5, infecting most of the isolates at the study site, was characterized with minor sequence variations between and within clones, resembling the polymorphisms observed earlier in partitiviruses and HetRV6 strains at the same forest site (Vainio et al., 2015a).

The ssRNA virus community showed similar features as detected earlier for dsRNA viruses in *H. parviporum* and *H. annosum* (Vainio et al., 2015a; Hyder et al., 2018), that is, variation in the viral content among isolates of a specific clone as well as disappearance and appearance of virus infections at certain sampling points during time. In addition to *Heterobasidion*, irregularity in the virus distribution within fungal clones has been reported for *C. parasitica* (Hoegger et al., 2003), *Helicobasidium mompa* (Ikeda et al., 2005), *R. necatrix* (Yaegashi et al., 2013), as well as *Lactarius rufus* and *Lactarius tabidus* (Sutela and Vainio, 2020), for instance. The patchy virus distribution within a clone may arise if a clone comes disconnected from the mycelial network or may represent the uneven mosaic-like distribution of viruses among different zones of the mycelium (e.g., Sasaki et al., 2007; Kim et al., 2013). As the partiti- and orthocurvulavirus infections were previously shown to increase over time (Vainio et al., 2015a), we hypothesized an increase in ssRNA viral infections and co-infections in the isolates of the study site after 7 years. Indeed, we found that single *H. parviporum* isolates harbored more viral infections in 2012 than in 2005, although the cultivation counts of hosts isolated year 2005 and 2012 may differ and thus contribute to the results obtained.

The highly stable and gradually expanding virus community at the site seems to be related to the host biology, i.e., presence of long-living clonal host individuals, and is very different from the annually variable virus community seen in the plant pathogen *S. sclerotiorum* infecting annual crops (Jia et al., 2021). The detailed virus population analysis conducted in this study combined with knowledge of the host genotype is unique for fungal botourmia- and ambi-like viruses, and gives new insight on the dispersal of these relatively recently discovered virus groups within and among conspecific host strains.

## Data Availability Statement

The sequences of virus genomes are available in the GenBank database under the accession numbers MZ502381-90 and the RNA-Seq reads deposited in the SRA and can be accessed through PRJNA738388.

## Author Contributions

EV: conceptualization, project administration, and funding acquisition. EV, SS, and TP: formal analysis and investigation. EV and SS: writing original draft preparation. All authors have read and agreed to the published version of the manuscript.

## Conflict of Interest

The authors declare that the research was conducted in the absence of any commercial or financial relationships that could be construed as a potential conflict of interest.

## Publisher’s Note

All claims expressed in this article are solely those of the authors and do not necessarily represent those of their affiliated organizations, or those of the publisher, the editors and the reviewers. Any product that may be evaluated in this article, or claim that may be made by its manufacturer, is not guaranteed or endorsed by the publisher.

## Footnotes

1
https://talk.ictvonline.org/

2
https://talk.ictvonline.org/files/proposals/taxonomy_proposals_fungal1/m/fung01/12412
