# Supplementary material for: Discovery and Community Dynamics of Novel ssRNA Mycoviruses in the Conifer Pathogen Heterobasidion parviporum
Source: Front Microbiol. 2021 Nov 24;12:770787. doi: 10.3389/fmicb.2021.770787 (PMC8652122; doi:10.3389/fmicb.2021.770787)
Supplement: Supplementary file 1 [file Data_Sheet_1.pdf]

Supplementary Material

1 Supplementary Figures

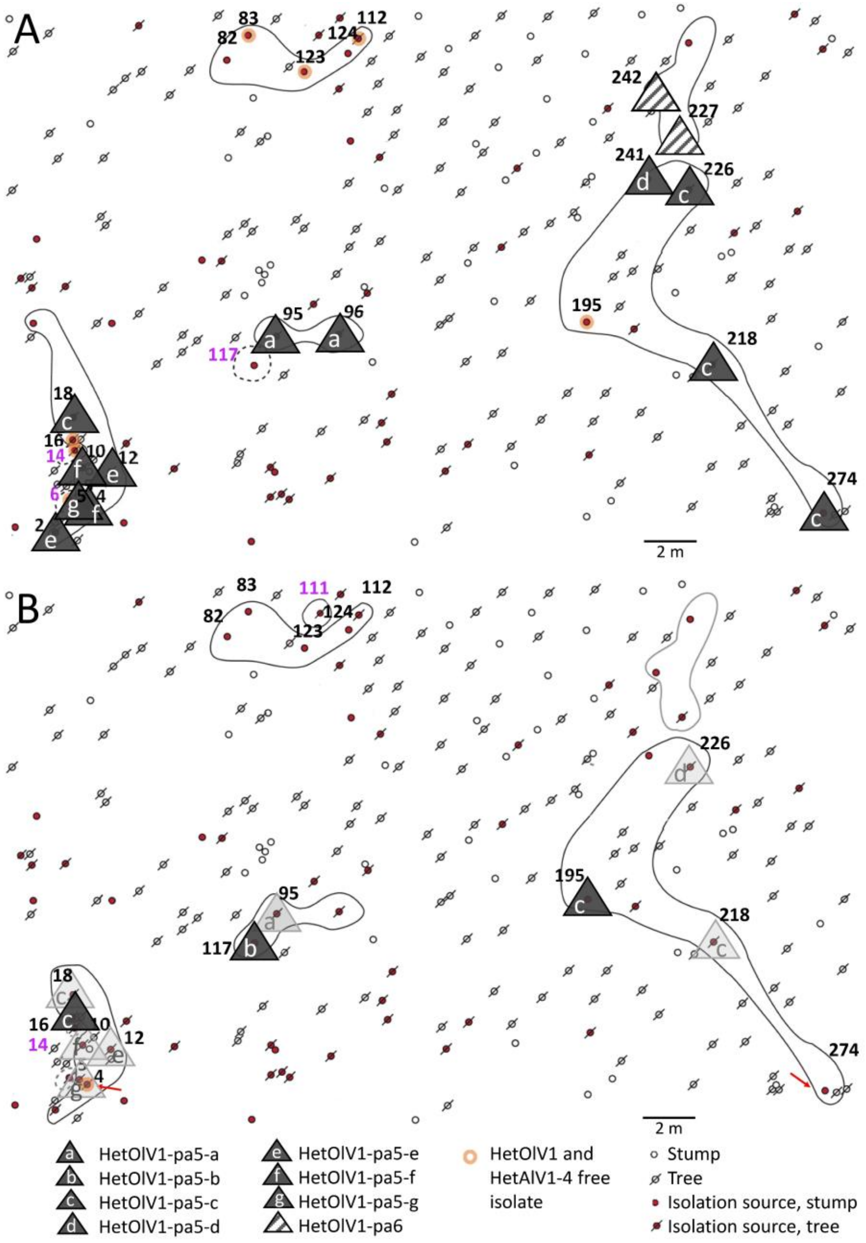

Supplementary Figure 1. Spatial distribution of *H. parviporum* isolates and virus strains and variants of Heterobasidion ourmia-like virus 1 (HetOlV1). The isolation sources refer to individual stumps and trees with *H. parviporum* infection 2005 (A) and resampled 2012 (B). *H. parviporum* isolation sources included in the analyses are indicated with numbers black font = clone includes more than single isolate; purple = clone includes only one isolate) and clones marked with circles (solid line = heterokaryon; dashed line = homokaryon; black line = viable clone; grey line = clone not found in 2012). Red arrows indicate viral losses. Triangles in light gray (B, 2012 situation) indicate that the virus content remained the same as in 2005, and dark gray color indicate virus gains.

2 Supplementary Tables

Supplementary Table 1. Primers utilized in the current study

Target amplicon	Purpose	Trinity contig	Forward primer	Sequence of forward primer	Reverse primer	Sequence of reverse primer
HetOlV1	Screening of isolates	DN24714	7-TRI-DN24714OurmiaF	TTGGCGTCCCTTTCCCTAAT	7-TRI-DN24714OurmiaR	ACACGAGGAGTTCTTGAGGG
HetOlV1	Sanger sequencing	DN24714	7Het-Ourmia-BegF	AGTTCTACCTTCAGCTCCGG	7Het-Ourmia-MidR	CCGACCCCTTTTATCCCCTT
HetOlV1	Sanger sequencing	DN24714	7Het-Ourmia-MidF	CGGTGAAGACGACATTCTGAC	7Het-Ourmia-EndR	CGCACCCGTTGAAACCTCT
HetOlV1	Sequencing of the UTR	DN24714	7-TRI-DN24714Ourmia-RT-F	CCTTGTCCCAGCCAATAGGAAA	T4 RNA primer[a] or primer A[b]	
HetOlV1	Sequencing of the UTR	DN24714	7-TRI-DN24714Ourmia-RT2-F	CCGAGTCATCAACAACCATT	T4 RNA primer[a] or primer A[b]	
HetOlV1	Sequencing of the UTR	DN24714	T4 RNA primer[a] or primer A[b]		7-TRI-DN24714Ourmia-RT-R	CGAAAACAACGTTACCCTTGCG
HetOlV1	Sequencing of the UTR	DN24714	T4 RNA primer[a] or primer A[b]		7-TRI-DN24714Ourmia-RT2-R	CGTGGGATGCCAACTAATAG
HetOlV1	Sequencing of the UTR	DN24714	T4 RNA primer[a] or primer A[b]		7HET-Ourmia-RTR3	GCCCAGTGGAAACCTTTGAT
HetPV21 RNA1	Screening of isolates		7-C114&135PearRdRpF	GAGAGAGAGGAGGAGCGTTC	7-C114&135PearRdRpR	TCGAAGTCCTGGCAAGCTTA
HetPV21 RNA1	Sequencing of the UTR		Pear_RdRP_RT_F	GAAACGCTTGAGCGCGTGAT		
HetPV21 RNA1	Sequencing of the UTR		T4 RNA primer[a]		Pear_RdRP_RT_R	CGAACGCTCCTCCTCTCTCT
HetPV21 RNA2	Screening of isolates	DN15879	7-TRI-DN15879PearCPF	AACGACTTGCTTTGCGATCA	7-TRI-DN15879PearCPR	AGTGGAGTCGTCAGTTGGAG
HetPV21 RNA2	Sequencing of the UTR	DN15879	Pear_CP_RT_F	TTGAAGTCCCGTGTCAAAGCC	T4 RNA primer[a]	
HetPV21 RNA2	Sequencing of the UTR	DN15879	Pear_CP_RT2_F	TACTTCTGAAGCACCCAAGA	T4 RNA primer[a]	
HetPV21 RNA2	Sequencing of the UTR	DN15879	Pear_CP_RT_F2	CGACACACTTCCCTCTTCCC	T4 RNA primer[a]	
HetPV21 RNA2	Sequencing of the UTR	DN15879	T4 RNA primer[a]		Pear_CP_RT_R	CTGCTTGAGACTTGGAGGCG
HetNlV1 RNA1	Screening of isolates	DN23600	7-HET-DN23600-F2	ATCGCGAGTACATGTGGGAT	7-HET-DN23600-R2	CTCAAGAGCCAATGACGTCG
HetNlV1 RNA1	Sanger sequencing	DN23600	7Het-narna-BegF	TGCTAGCCTGCCCTCTTATG	7Het-narna-MidR	GGAGGTAAGGGTTGGTAGGC
HetNlV1 RNA1	Sanger sequencing	DN23600	7Het-narna-MidF	CGCGAGGTCCATGGTATAGT	7Het-narna-EndR	TAAAGCGCCTGTGCTACCTT
HetNlV1 RNA1	Sequencing of the UTR	DN23600	7HET-Narna-RTF2	AGTGGGACTGGTTGGAGC	T4 RNA primer[a]	
HetNlV1 RNA1	Sequencing of the UTR	DN23600	T4 RNA primer[a]		7HET-Narna-RTR2	TCATAAGAGGGCAGGCTAGC
HetNlV1 RNA1	Sanger sequencing	DN23600	7Het-narna-EndF	CACCTATACCCTTCATCCCCA		
HetNlV1 RNA1	Sanger sequencing	DN23600			7Het-narna-EndR2	ATGCCCAAACTATACGCTGC
HetNlV1 RNA2	Sanger sequencing	DN23310	7H_Nar2_BegF	AGTTCGCTTGCAGCACATTA	7H_Nar2_MidR	TGTGTCGTCGGACTCAAATC
HetNlV1 RNA2	Sanger sequencing	DN23310	7H_Nar2_MidF	ATTTGACGAAGCCGAGAAAA	7H_Nar2_EndR2	CGGTACTGCCCCACTTCTTA
HetNlV1 RNA2	Sanger sequencing	DN23310			7H_Nar2_BegR	GCTTCGGTGGTTCAGATGTT
HetNlV1 RNA2	Sanger sequencing	DN23310	7H_Nar2_BegF2	GAGTCCACGGTCGAAGTACC		
HetNlV1 RNA2	Sanger sequencing	DN23310			7H_Nar2_EndR	ACCACCAAGCATCATTCCAT
HetNlV1 RNA2	Sanger sequencing	DN23310	7H_Nar2_EndF	CAACCAGTACGTCGACCACA		
HetNlV1 RNA2	Sequencing of the UTR	DN23310	7H_Nar2_RTF	TGCTCCAGATGATGAACGAG	T4 RNA primer[a]	
HetNlV1 RNA2	Sequencing of the UTR	DN23310	T4 RNA primer[a]		7H_Nar2_RTR	TAGGGCAGCTGACTTGGTCT
HetMV3	Screening of isolates	DN25156	MV3ScRForM	TAATGGAGGATGGCATTGGT	3rdMVEndR3	CCCACAGTTAAATCGTCCCC
HetMV3	Sanger sequencing	DN25156	3rdOrfEndF2	TCTCCTAGAGTAGCTGCGG	3rdMVEndR3	CCCACAGTTAAATCGTCCCC
HetAlV1	Screening of isolates	DN17906	7HET_AS1_F	AGACTCGTGGTGTCGGCTTGG	7HET_AS1_MidR	CCGATCCGGAATAACCACTA
HetAlV1	Sanger sequencing	DN17906	7HET_AS1_EndF	TCCAAGGCAGAGAAGCCTAA	7HET_AS1_MidR	CCGATCCGGAATAACCACTA
HetAlV1	Sanger sequencing	DN17906	7HET_AS1_F	AGACTCGTGGTGTCGGCTTGG	7HET_AS1_EndR	TCAATACCCACACCTGCAAA
HetAlV1	Sanger sequencing	DN17906	7HAS1_ORFA_MidF	AAGGGCCCATACTCTCCAAG		
HetAlV1	Sanger sequencing	DN17906	7HAS1_ORF2_MidF	GGGACAGAAGTTCCAGACCA		

HetAlV2	Screening of isolates	DN25337	7HET_AS2_F	7CTTGTACCCGGTCTCGTGAT	7HET_AS2_MidR	TTCATTGTGGACTCGCCATA
HetAlV2	Sanger sequencing	DN25337	7HET_AS2_EndF	GACCCTGAACGGAAAATTCA	7HET_AS2_MidR	TTCATTGTGGACTCGCCATA
HetAlV2	Sanger sequencing	DN25337	7HET_AS2_MidF	GGTCTGTGGTGCCTGTCTTT	7HET_AS2_EndR	CTATGGACGCCCTACCCTTT
HetAlV2	Sanger sequencing	DN25337	7HAS2_ORFA_BegR	GGATCGCCTTTTACCTGCAG		
HetAlV2	Sanger sequencing	DN25337	7HAS2_ScrF	GGTGTCGAAGGGAGGATGAA	7HAS2_ScrR	CCAATGCCCGAAACCATGAA
HetAlV2	Sanger sequencing	DN25337			7HAS2_ORFA_MidR	AGCTGCATGTTTCTTCATGG
HetAlV2	Sanger sequencing	DN25337	7HAS2_ORFA_EndF	TGTACGCCACCACAGATGTT		
HetAlV2	Sanger sequencing	DN25337			7HAS2_ORF2_MidR	CCAGGATAGGAGTGGGTCAA
HetAlV2	Sanger sequencing	DN25337	7HAS2_ORF2_MidF	TGGACCATCAGAAGGTGTTG		
HetAlV3	Screening of isolates	DN20679	7HET_AS3_F	TCGAAACCCTATGTCCTTCG	7HET_AS3_MidR	TGAGTGTTCTGTGCCTCCAG
HetAlV3	Sanger sequencing	DN20679	7HET_AS3_EndF	ATGGGTTTGTTCACGTGGTT	7HET_AS3_MidR	TGAGTGTTCTGTGCCTCCAG
HetAlV3	Sanger sequencing	DN20679	7HET_AS3_MidF	GACCCGTGTGAATGTCAAAA	7HET_AS3_EndR	TCAACAGAGCATCTCGTTCCT
HetAlV3	Sanger sequencing	DN20679	7HAS3_ORFA_EndF	AGGAAGACAGGTGGTTAGGC		
HetAlV3	Sanger sequencing	DN20679	7HAS3_ORFA_MidF	GCGCCAGTTTGTACGATGAA		
HetAlV3	Sanger sequencing	DN20679			7HAS3_ORFA_BegR	GGGCGTACTGTTTGACTTCC
HetAlV3	Sanger sequencing	DN20679			7HAS3_ORFA_MidR	CGAGAAGATTCCAGCTCCCA
HetAlV3	Sanger sequencing	DN20679			7HAS3_ORF2_MidR	CATACGCTTTCCCCACATTT
HetAlV3	Sanger sequencing	DN20679	7HAS3_ORF2_MidF	ACGCATGCCTCTAAACTGCT		
HetAlV3	Sanger sequencing	DN20679			7HAS3_ORF2_MidR2	TGGTCGAATGTCACCATGTC
HetAlV3	Sanger sequencing	DN20679	7HAS3_ORF2_EndF	CAACAGACGCTCTTGGAAAA		
HetAlV4	Screening of isolates	DN19684	7HET_AS4_F	ATGGCCACCTGCTTTAACAC	7HET_AS4_MidR	TCACTTCGATCTTGCCCTCT
HetAlV4	Sanger sequencing	DN19684	7HET_AS4_EndF	ATGGACTCGGTCTGCTTTTG	7HET_AS4_MidR	TCACTTCGATCTTGCCCTCT
HetAlV4	Sanger sequencing	DN19684	7HET_AS4_MidF	TCCCAGAAAGCGATAATTCG	7HET_AS4_EndR	TTCGATACGGTTGTTTGCAG
HetRV6	Screening of isolates	DN25035	CurF1Aus	TTGAATCACCTGGACCGTTT	CurRe2	CATCAACCCATTATCCAGGT
HetRV6	Sanger sequencing	DN25035	HV6BegF1	CAATAAAGAAGGGACCTCAG	HV6EndR2	GCAGACCCGATGGAAAGA
HetRV6	Sanger sequencing	DN25035	HetRV6_RdRp_2019For	CTCGACAAGGTGCGGTTC		
HetRV6	Sanger sequencing	DN25035	HetRV6_RdRp_2019For2	GAGTTCGTCTTCCAGGGGTT		
HetRV6	Sanger sequencing	DN25035			HetRV6_RdRp_2019Rev	ATCAACTTCTCCCACGCCTC
HetPV4 RNA2	Sanger sequencing	DN28315	Rt349c_CoatFor	TTGTCAACAACTCAGTGTCA	Rt349c_CoatRev	GGGAATCTTCGACGAATTTG
HetPV4 RNA2	Sanger sequencing	DN28315	Rt349c_CoatFor2	CGTCCACCCACAATATCAAA	Rt349c_CoatRev2	GAAGAGGGAGAGAACGACTA
HetPV4 RNA2	Sequencing of the UTR	DN28315	Rt349c_CoatRTFor	CCCAACGTTCGGATAATTTC	T4 RNA primer[a]	
HetPV4 RNA2	Sequencing of the UTR	DN28315	Rt349c_CoatRTFor2	CTCGGACACAGCAGGATTTC	T4 RNA primer[a]	
HetPV4 RNA2	Sequencing of the UTR	DN28315	T4 RNA primer[a]		Rt349c_CoatRTRev	ATCGGAGGATGTGGTAGTAG
HetPV4 RNA2	Sequencing of the UTR	DN28315	T4 RNA primer[a]		Rt349C_coatRTREV2019	GGTAGGAGCCGCATTCTTTG

[a]Modified after Lambden et al. (1992) by Tuomivirta and Hantula (2003).

[b]Adapter sequence as described in Attoui et al. (2000).

Supplementary Table 2. Variant calling with Geneious[a] for selected viruses hosted by *H. parviporum* isolates included to RNA-Seq library.

Target virus[b]	Length (nt)	Sequence variants[d]	Reads[e]	Variant call parameters[g] Variant min frequently[h]	SNP sites identified		SNPs not found	
					Variant call[i]	Sanger seq[j]	Variant call[k]	Sanger seq[l]
HetNlV1-pa1[c]	1,931	2	88,303[f]	15%	58	58	1	0
HetOlV1-pa1-a	967	6	38,854[f]	2.5%	59	58	0	1
HetAlV2-pa1-a	929	3	221,639[f]	10%	24	24	0	0
HetAlV3-pa1-a	630	4	126[f]	5%	19	24	4	1
HetMV3-pa1-4	1,451	2	746,895[f]	5%	80	78	1	3
HetRV6-pa33[c]	1,821	7	10,635[f]	7.5%	138	138	0	0

[a]Geneious 10.2.6 or Prime
[b]The virus strain/variant utilized in the mapping and variant call
[c]RNA1 was utilized in the mapping and variant call
[d]Number of sequence variants (strains and variants) found present in the RNA-Seq library
[e]Mapping conducted with Geneious for RNA Seq with medium-low sensitivity (gaps allowed, word length 18, words repeated more than 12 times ignored, 20% maximum mismatches allowed per read)
[f]Total number or raw reads mapped against the partial virus sequence
[g]Geneious variant call was conducted with default parameters (10e-5 = maximum variant P-value; 10e-6 = minimum strand-bias P-value) except for variant frequently
[h]Minimum variant frequently
[i]SNP sites detected with variant call
[j]SNP sites detected aligning Sanger confirmed sequences of virus sequence variants
[k]Number of SNPs not identified with variant call but found present when aligned Sanger confirmed sequences were examined
[l]Number of SNPs not found present in the alignment of Sanger confirmed sequences but identified using variant call
HetNlV1: Heterobasidion narna-like virus 1; HetOlV1: Heterobasidion ourmia-like virus 1; HetAlV2: Heterobasidion ambi-like virus 2; HetAlV3: Heterobasidion ambi-like virus 3; HetMV3: Heterobasidion mitovirus 3; HetRV6: Heterobasidion RNA virus 6

3 Supplementary References

Attoui, H., Billoir, F., Cantaloube, J. F., Biagini, P., de Micco, P. & de Lamballerie, X. (2000). Strategies for the sequence determination of viral dsRNA genomes. J Virol Methods 89, 147-158. doi: 10.1016/s0166-0934(00)00212-3

Lambden, P.R., Cooke, S.J., Caul, E.O., Clarke, I.N. (1992). Cloning of noncultivatable human rotavirus by single primer amplification. J. Virol. 66, 1817-1822. doi: 10.1128/JVI.66.3.1817-1822.1992

Tuomivirta, T.T., and Hantula, J. (2003b). Gremmeniella abietina mitochondrial RNA virus S1 is phylogenetically related to the members of the genus Mitovirus. Arch. Virol. 148, 2429-2436. doi: 10.1007/s00705-003-0195-5
